# Non-Coding RNAs as Novel Regulators of Neuroinflammation in Alzheimer’s Disease

**DOI:** 10.3389/fimmu.2022.908076

**Published:** 2022-06-02

**Authors:** Yuqing Liu, Xin Cheng, Hongli Li, Shan Hui, Zheyu Zhang, Yang Xiao, Weijun Peng

**Affiliations:** ^1^ Department of Integrated Traditional Chinese and Western Medicine, The Second Xiangya Hospital, Central South University, Changsha, China; ^2^ National Clinical Research Center for Mental Disorder, Changsha, China; ^3^ Department of Geratology, Hunan Provincial People’s Hospital, The First Affiliated Hospital of Hunan Normal University, Changsha, China; ^4^ National Clinical Research Center for Metabolic Diseases, Key Laboratory of Diabetes Immunology, Ministry of Education, Changsha, China; ^5^ Department of Metabolism and Endocrinology, The Second Xiangya Hospital, Central South University, Changsha, China

**Keywords:** non-coding RNAs, Alzheimer’s disease, neuroinflammation, regulators, therapeutic targets

## Abstract

Alzheimer’s disease (AD) is one of the most common causes of dementia. Although significant breakthroughs have been made in understanding the progression and pathogenesis of AD, it remains a worldwide problem and a significant public health burden. Thus, more efficient diagnostic and therapeutic strategies are urgently required. The latest research studies have revealed that neuroinflammation is crucial in the pathogenesis of AD. Non-coding RNAs (ncRNAs), including long noncoding RNAs (lncRNAs), microRNAs (miRNAs), circular RNAs (circRNAs), PIWI-interacting RNAs (piRNAs), and transfer RNA-derived small RNAs (tsRNAs), have been strongly associated with AD-induced neuroinflammation. Furthermore, several ongoing pre-clinical studies are currently investigating ncRNA as disease biomarkers and therapeutic interventions to provide new perspectives for AD diagnosis and treatment. In this review, the role of different types of ncRNAs in neuroinflammation during AD are summarized in order to improve our understanding of AD etiology and aid in the translation of basic research into clinical practice.

## 1 Introduction

Alzheimer’s disease (AD) is a degenerative disease characterized by progressive deterioration of memory and cognitive function, leading to loss of autonomy and accounting for at least two-thirds of dementia cases in patients aged ≥65 years (1, 2). More than 3 out of every 10 elderly people aged >85 years suffer from AD worldwide (3). Age and gender both influence AD occurrence, with approximately two-thirds of AD patients being women (4).Gonadal hormones, environment, society, and culture have different effects on women and men, and there is growing evidence that gender influences the cause, presentation, and treatment outcomes of many diseases. At present, there are about 50 million AD patients in the world, with about one-third of these cases occurring in China. However, research on the mechanisms driving the pathogenesis of AD is far behind expectations.

**Figure 1:**
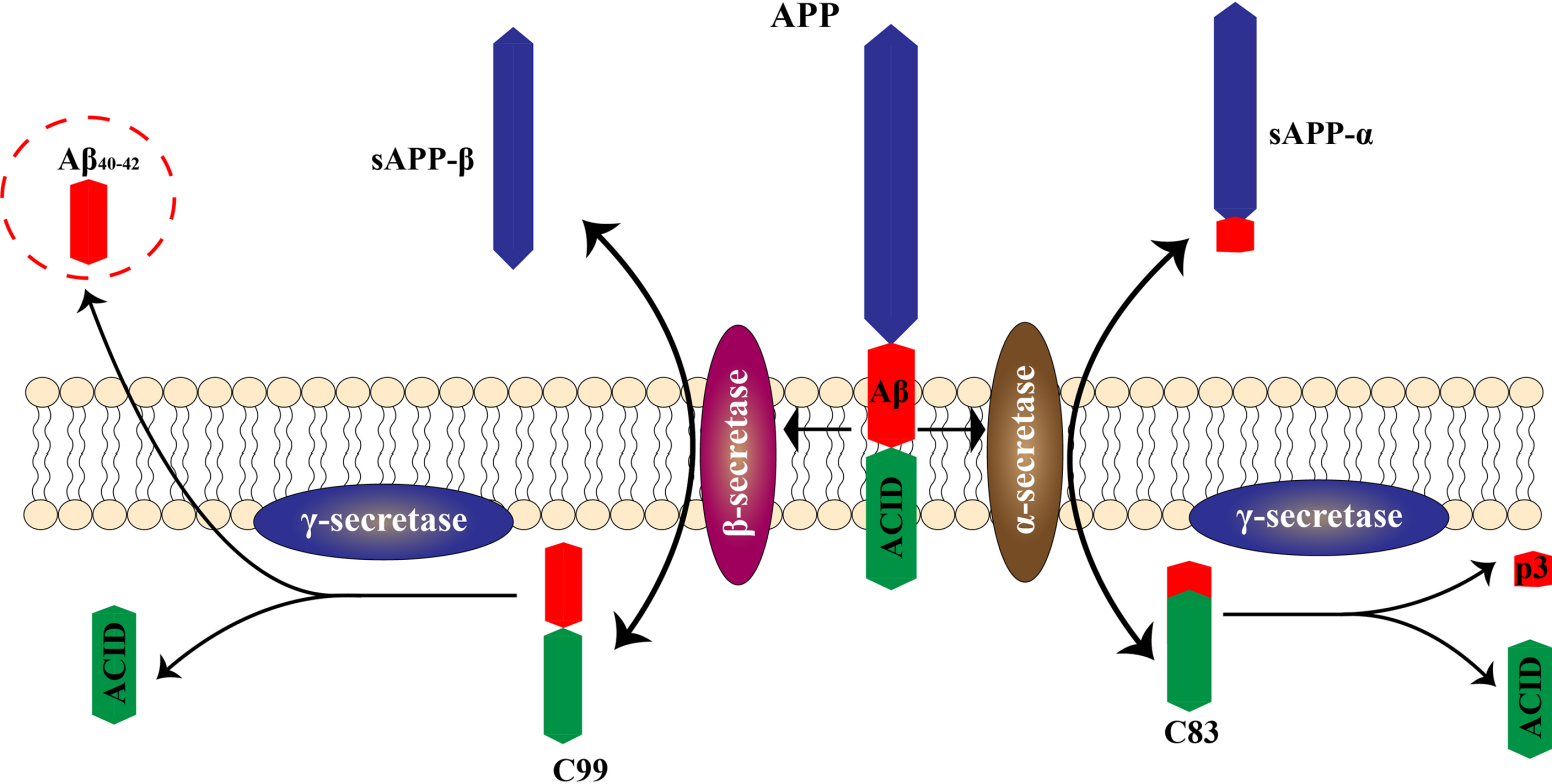
Pathological and physiological APP metabolism. APP is cleaved by two enzymes, α secretase and β secretase, resulting in different outcomes. α secretase excises the amyloid region, preventing Aβ production. The co-produced C83 protein and sAPPα enter the cytoplasm without causing neurotoxicity. The β-secretase cleavage site bypasses the amyloid region, producing sAPPβ and C99. Further processing of C99 leads to production of intracellular binding domain of amyloid precursor protein (AICD) fragments and Aβ. Aβ molecules bind each other to form oligomers, eventually forming fibers that are neurotoxic.

Several hypotheses have been proposed to explain the actual pathological development of AD (5). One of the earliest of these was the cholinergic hypothesis. Characteristic symptoms, such as cognitive impairment, were initially thought to be caused by cholinergic deficiency due to cholinergic neuron degeneration. Schumacher et al. (6) found that patients with mild cognitive impairment exhibited degeneration of the Meynert basal cholinergic nucleus accompanied by an early reduction in the integrity of white matter projections originating in this structure. This degeneration of the Meynert’s basal cholinergic nucleus was positively correlated with cognitive impairment. With advancing research, two proteins deposited in the brain—beta-amyloid (Aβ) and hyperphosphorylated tau protein—were identified and are considered to play crucial roles in the development of AD (7). Aβ is abnormally deposited in the brain of AD patients, forming plaques that attenuate neuronal function. Cleavage of the transmembrane amyloid precursor protein (APP) produces Aβ (8). Senile plaques are formed when Aβ is deposited outside of the cell (9, 10) (**Figure 1**). Aβ is a neurotoxic protein that plays a key role in neuronal hypofunction and the progression of AD. Hyperphosphorylated tau protein aggregates to form insoluble fibrous tangles that block synaptic transmission (11). The progressive development of neuritis plaques is a crucial driving factor of AD.

More recently, neuroinflammation has emerged as another key cause of AD. Many studies have tentatively shown a persistent inflammatory response within the brain of AD patients (12). Aβ may act as a neurotoxic and inflammation-related protein that activates microglia and astrocytes and promotes the inflammatory response (13, 14). In addition, glial proliferation has been found clustered around pathological neurofibrillary tangles. Furthermore, microglia release inflammatory cytokines in response to Aβ (15–17). Thus, the occurrence and progression of AD may be due to persistent inflammation in the brain that leads to nerve damage and neuronal death (18, 19). Studies have shown that the neuroinflammatory response plays a dual role in the pathological development of AD (20). On one hand, inflammation promotes Aβ phagocytosis by microglia to reduce Aβ deposition. On the other hand, excessive inflammation may cause irreversible damage to nerve tissue (3). This dual effect may be an important manifestation of Aβ neurotoxicity and may contribute to the development and progression of AD.

## 2 Role of Neuroinflammation in the Course of AD

**Figure 2:**
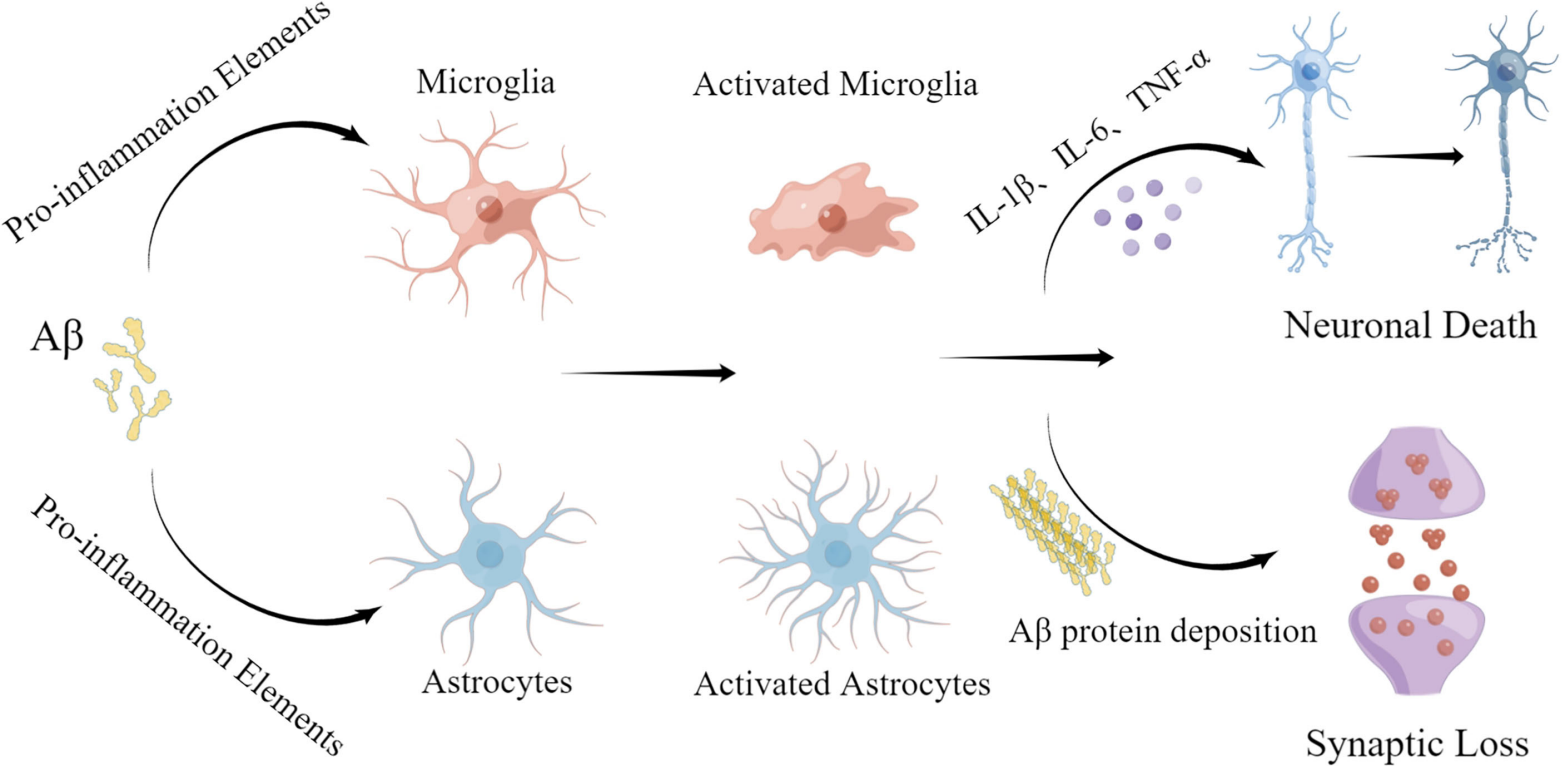
Roles of microglia and astrocytes in AD. The neurotoxic effects of Aβ protein promote activation of microglia and astrocytes, leading to a neuroinflammatory response. Excessive activation promotes deposition of Aβ protein, leading to further nerve damage.

Neuroinflammation is regarded as an inflammatory response occurring in the central nervous system that is triggered by ischemia, hypoxia, shock, or other injuries. Inflammation in nerve tissue appears to play a dual role in the development of AD, counteracting neurotoxic effects in the initial stages of the acute response and becoming harmful in later stages that are characterized by a persistent inflammatory response (21). Activated microglia and astrocytes are central to neuroinflammation (**Figure 2**). In AD, it has been hypothesized that the presence of Aβ is the main driver of microglial activation. Moreover, activated microglia respond to Aβ, and numerous studies have shown that activated microglia engulf Aβ (20). This increased immune response leads to Aβ clearance early in the onset of AD (22). However, continued immune response activation or overactivation leads to reduced microglia binding and phagocytosis of Aβ, as well as reduced microglia antibody-degrading enzyme activity, resulting in decreased phagocytosis of Aβ plaques. Although some microglia functions are inhibited during continued activation or overactivation, the ability of microglia to produce pro-inflammatory cytokines is unaffected, leading to neurodegeneration and additional microglia activation (3, 12).

Microglia and astrocytes mainly release interleukin 1 beta (IL-1β), interleukin 6 (IL-6), tumor necrosis factor (TNF), nitric oxide (NO), and reactive oxygen species (23). Damaged capillary endothelial cells and invading blood cells may also promote neuroinflammation, especially when the blood-brain barrier (BBB) is continuously damaged. When the BBB is disrupted, pro-inflammatory molecules are more prone to contact nerve tissues, causing synaptic damage and neuronal death (24, 25). TNF binds to the tumor necrosis factor receptor 1 (TNFR1), leading to caspase 8 aggregation and neuronal death (26). The complement system is also activated as part of the inflammatory response. Once activated, it promotes phagocytosis by microglia. Thus, its overactivation may lead to inappropriate synaptic pruning. In neuroinflammatory responses, anti-inflammatory factors such as IL-1 receptor antagonists, interleukin 4 (IL-4), interleukin 10 (IL-10), and interleukin 11 (IL-11) are also produced, possibly as part of a protective mechanism against neuroinflammatory overactivation (27). In conclusion, in neurodegenerative diseases, neuroinflammation is often a persistent process that causes irreversible damage and is considered to be an important driver of the disease state.

A 2019 meta-analysis (28) of 170 studies that measured peripheral or cerebrospinal fluid inflammatory markers combined results from 9,842 AD patients, 3,526 mild cognitive impairment (MCI) patients, and 9,002 control patients and found statistically significant changes in both blood and cerebrospinal fluid inflammatory markers in AD patients. These results indicate the presence of peripheral and neuroinflammatory responses in patients with AD. Studies have shown that peripheral and cerebrospinal fluid inflammation may mediate early cognitive impairment in neurodegeneration. Possible pathogenic processes that contribute to the progressive development of AD include inhibition of angiogenesis and neurotrophic and neuroprotective mechanisms, induction of neuron loss, and activation of myelin sheath injury (29). Although inflammation has a protective effect on the body, excessive inflammation may lead to or contribute to tissue damage and disease pathology. Studies have found that the occurrence, transmission, and resolution of inflammatory responses in central nervous system diseases depend on soluble factors including cytokines and various ncRNAs. Microglia, for example, participate in the inflammatory reaction by releasing vesicles enriched in IL-1β and miR-155 or TNF-α and IL-6 in response to lipopolysaccharides (LPS) stimulation. In addition, miR-146a is secreted by hippocampal-located exosomes in AD mouse and human brains and binds to the toll-like receptor (TLR) to activate the pro-inflammatory response and cytokine release, and this response is related to disease severity (30). Most studies believe that ncRNA plays a dual role in AD. Maintaining the balance between the beneficial and harmful effects of exosomes on neuropathological progression and the ability to exogenously alter this balance for the treatment of AD remains to be determined (31–33).

## 3 Role of Non-Coding RNAS in AD Neuroinflammation

ncRNAs are a widely diversified family of non-protein-coding transcripts found in various tissues that comprise at least 98% of the total genome, based on human transcriptome sequencing (34). ncRNAs regulate heredity, epigenetic inheritance and translation (35), and can be divided into long non-coding RNA (lncRNA, >200 nt), small ncRNA (sncRNA, < 200 nt), and circRNA. Moreover, sncRNA can be further classified as microRNA (miRNA), piwi-associated small RNA (piRNA), or transfer RNA-derived small RNA (tsRNA), among others. ncRNA networks regulate a variety of physiological processes, such as gene transcription, mRNA translation, and protein modification through their unique structural and functional roles (36). Pathological neuroinflammation underlies many central nervous system diseases, including AD (20). Determining the specific mechanisms that initiate and maintain neuroinflammation will undoubtedly help slow the progression of AD and allow development of new treatment strategies. ncRNA is one of the important molecules that regulate neuroinflammatory signals (37). In fact, specific ncRNAs may be common to many diseases in which they play a crucial role in controlling inflammation (38). While some ncRNAs may be disease-specific, others exert cumulative effects by interacting with multiple ncRNAs to influence neuroinflammation during disease. In neuroinflammatory diseases, ncRNAs also circulate in biological fluids and exhibit specific patterns of altered expression. Identifying the specific mechanisms of ncRNA action in these diseases, including AD, may allow for the development and research of their diagnostic and therapeutic applications.

### 3.1 MicroRNAs (MiRNAs) in AD

miRNAs are small single-stranded ncRNAs, ranging from 19–25 nucleotides long, that post-transcriptionally regulate gene expression in plants and animals by binding to the 3’ untranslated region (3’UTR) of their mRNA target (39). Data suggest that miRNAs contribute to synaptic plasticity, neuronal differentiation, and development. In addition, >50% of mRNAs are predicted to host miRNAs, enabling them to regulate most biological processes, including neurogenesis, apoptosis, inflammation, and oxidative stress (40). Studies have found that miRNA is specifically expressed in immune cells, where it may regulate the activation and function of these immune cells. Later, it was found that miR-155, miR-146, and miR-223 can bind TLRs to regulate the acute inflammatory response (41). There is evidence that miRNAs play a central role in regulating AD progression. Furthermore, many miRNAs target several genes related to post-AD inflammation. Transcription of several important miRNAs involved in neuroinflammation is regulated by nuclear factor-kappa B (NF-кB). NF-кB is an immune- and stress-induced transcription factor (42). Acetylcholine blocks inflammation-induced NF-кB activation under physiological conditions, which is an important pathway in miRNA-related neuroinflammatory regulation (43, 44).

#### 3.1.1 MiR-155

**Figure 3:**
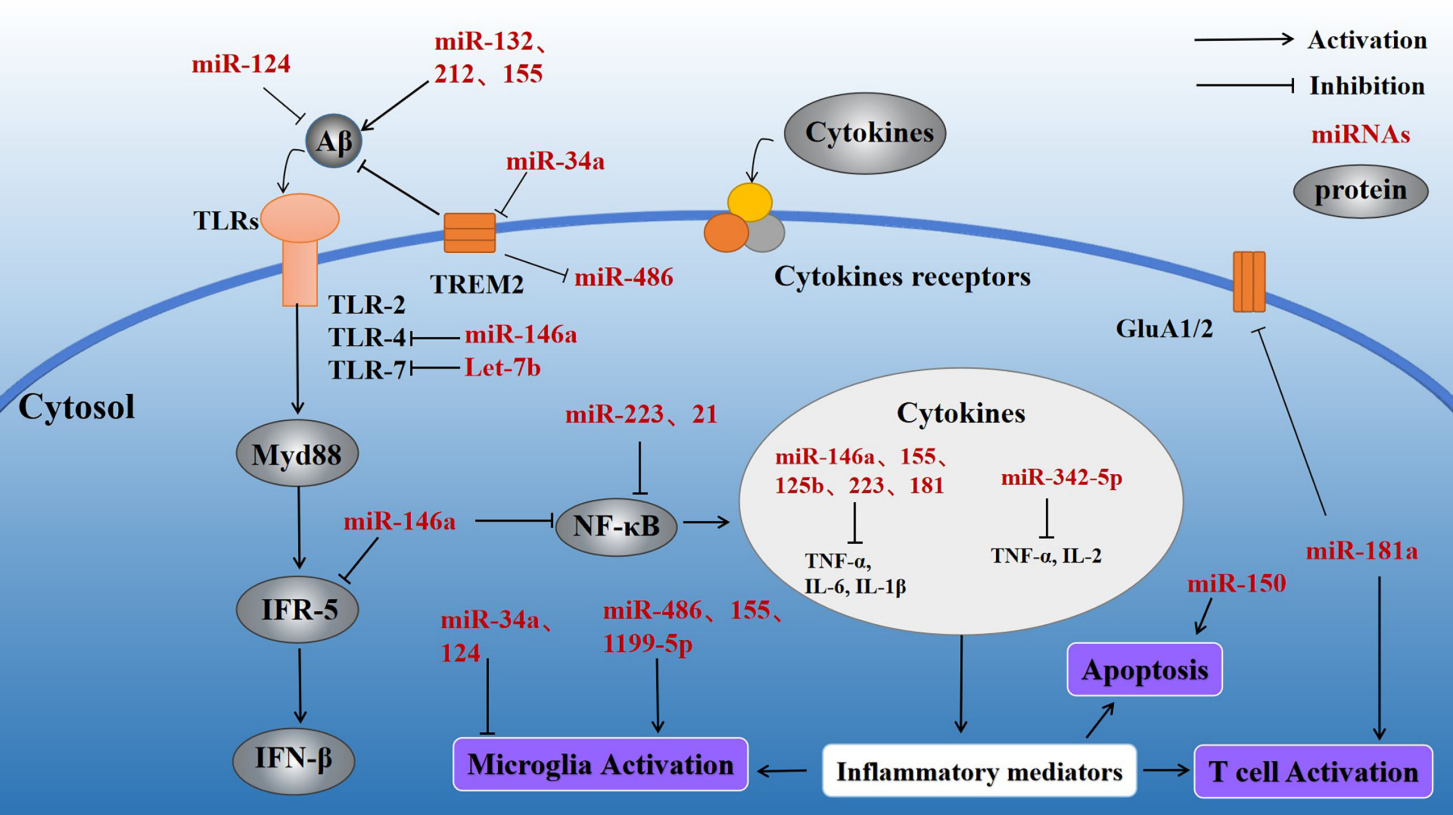
Effect of inflammatory miRNAs on AD inflammation. Multiple inflammatory miRNAs may play a synergistic or antagonistic role in different inflammatory pathways. For example, mir-146a targets the MyD88-related pathway by binding to TLR-4. At the same time, NF-κB is inhibited, preventing release of inflammatory factors. Meanwhile, miR-155 and miR-181a regulate a variety of inflammatory mediators. Microglial activation is important in various inflammatory processes and is stimulated by miR-34a and miR-486. Apoptosis, as the terminal link in the inflammatory response, also plays a significant part in the progression of AD and is regulated by miR-150.

miRNA-155 is a multifunctional miRNA with a unique expression profile that is related to hematopoietic, inflammatory, and immune processes (45). Clinical trials have shown that miR-155 regulation may be efficacious in treating diseases such as cancer (46). Experiments using target site blockers and RNA immunoprecipitation have shown that miR-155 binds to peptidylarginine deiminase type4 (PAD4) to regulate PAD4 transcription. Targeting miR-155 may help inhibit overproduction of neutrophil extracellular traps (NETs) in inflammatory diseases (47). In central neurodegenerative diseases, pro-inflammatory miR-155 overexpression reduces Aβ_40-42_ catabolism (48) (**Table 1**). In the mouse brain, miR-155 alters the BBB permeability in neuroinflammatory disorders in the central nervous system by regulating cell-interacting molecules. Moreover, miR-155 is involved in T cell immune function and increases formation of inflammatory cytokines, such as IL-6 and interferon-β (IFN-β) (49) (**Figure 3**). Further studies involving the regulation of miR-155 levels may promote innovative, effective therapies for AD.

**Table 1:** The main inflammatory ncRNAs for AD.

Gene	Function in AD	Expression	Target gene
**MicroRNAs**			
miR-155	Induced by Aβ aggregation; promotes microglia and astrocyte activation; increases production of inflammatory mediators such as IL-6 and IFN-β	upregulated	*SOCS-1 ↓*
miR-34a	Synaptic deficits; downregulates the phagocytosis of both *TREM2* and microglia3	upregulated	*SYT1 ↓*
miR-486	Promotes microglia and astrocyte activation		
miR-124	Synaptic deficits; promotes polarization of M2 microglia	upregulated	*PTPN1 ↓*
miR-1199–5p	Induces microglial neuroinflammation		*ROCK1↓*
miR-15a	Regulates inflammatory responses and apoptosis	downregulated	*ERK1*
miR-132	Promotes Aβ production and Tau hyperphosphorylation	upregulated	*SIRT1 ↓*
miR-146a	Involved in the negative feedback regulation of NF-κB activation; attenuates astrocytic inflammation; induces TLR tolerance in macrophages	upregulated	*ROCK1 ↓*
miR-223	Promotes production of TNF-α, IL-6, and IL-1β; involved in the negative feedback regulation of NF-κB activation	downregulated	*NLRP3↓*
miR-125b	Promotes Tau hyperphosphorylation; increases the activities of TNF-α, IL-1β, and IL-6	upregulated	*SphK1 ↓*
miR-181a	Regulation of T-cell activation threshold; synaptic deficits	upregulated	*SIRT1↓*
miR-150	Affects formation of mature B cells	downregulated	
miR-342-5p	Synaptic deficits; promotes neuronal apoptosis; increases production of inflammatory mediators such as IL-2 and TNF-α	upregulated	*AnkG ↓*
**LncRNAs**			
MALAT1	Affects microglia activation; inhibits NF-κB signaling pathway; inhibits neuronal apoptosis	upregulated	*miR-125b↑*
NEAT1	Promotes microglia and astrocyte activation; increases production of inflammatory mediators such as IL-2 and TNF-α	upregulated	*miR-128p↑*
RP11-543N12.1	Induces apoptosis and inhibits proliferation in cell model of AD by targeting miR-324-3p	upregulated	*miR-324-3p↑*
MEG3	Inhibits PI3K/Akt pathway; inhibits neuronal apoptosis	downregulated	
ANRIL	Promotes inflammation and apoptosis; promotes neurite outgrowth by binding to miR-125a	upregulated	*miR-125a↑*
17A	Regulates inflammation by GABABR; promotes apoptosis	upregulated	*GABABR2 ↓*
MAGI2-AS3	Regulation of Aβ-induced neurotoxicity and neuroinflammation	upregulated	*miR-374b-5p↑*
TUG1	Regulates inflammatory responses and apoptosis		*miR-15a↑ ROCK1↑*
**CircRNAs**			
Circ_0000950	Synaptic deficits; promotes neuronal apoptosis; increases production of inflammatory mediators such as IL-2 and TNF-α	upregulated	*miR-103↑, PTGS2↑*
CIRS-7	Inhibits NF-κB signaling pathway	downregulated	*miR-7↑*
NF1-419	Decreases production of inflammatory mediators such as IL-6 and IFN-β	downregulated	*AP2B1*
HDAC9	Increases Aβ production	downregulated	*miR-138 ↑*

↓ represents down-regulated expression. ↑ represents up-regulated expression.

#### 3.1.2 MiR-34a

miR-34a is a tumor-suppressing transcript whose deletion has been associated with a variety of human cancers, including brain malignancies (50). Interestingly, miR-34a is highly expressed in the adult mammalian brain and may be closely related to neuronal survival. miR-34a is primarily involved in the TP53 tumor suppressor network. miR-34a transcriptional activation is promoted by TP53 and may lead to cell cycle arrest and apoptosis (51). Meanwhile, in the brain of AD patients, miR-34a targets several genes that maintain normal physiological activity of neural tissues, such as genes related to synaptic plasticity, nerve repair, and memory (52). miR-34a is upregulated in the brain of AD patients (**Table 1**), and bioinformatics/transfected luciferase reporter assays show that miRNA-34a targets 299 nucleotides in the trem2-mRNA-3’UTR, leading to downregulation of triggering receptor expressed on myeloid cells 2 (TREM2). TREM2 is a transmembrane sensing receptor that plays a significant role in Aβ clearance. Aβ_42_ activates microglia to enhance Aβ phagocytosis, whereas miR-34a inhibits TREM2 activation of microglial phagocytosis (53) (**Figure 3**), and bioinformatics/transfected luciferase reporter assays show that miRNA-34a targets 299 nucleotides in the trem2-mRNA-3’UTR, leading to downregulation of triggering receptor expressed on myeloid cells 2 (TREM2). TREM2 is a transmembrane sensing receptor that plays a significant role in Aβ clearance. Aβ_42_ activates microglia to enhance Aβ phagocytosis, whereas miR-34a inhibits TREM2 activation of microglial phagocytosis (54).

#### 3.1.3 MiR-486

miR-486-5p is a miRNA with broad bioregulatory functions that may serve as a potential biomarker for the diagnosis of various cancers, including chronic myeloid leukemia (CML) (55). miR-486-5p affects the angiogenic activity of endothelial cells by regulating its target gene, matrix metalloproteinase 19 (MMP19) (56). It has also been found that the severity of the inflammatory response in sepsis is positively correlated with miR-486-5p expression in cells, making miR-486-5p a potential diagnostic biomarker of sepsis (57). Overexpression of miR-486 has been observed in myocardial ischemia cell models (58). miR-486 may be an important regulatory factor of the inflammatory response (59) (**Table 1**). In neural tissues, miR-486 expression is regulated by TREM2 (60, 61), which acts to alter the miRNA structural activity and physiological functions. This process results in altered levels of protein kinase B (Akt) and promotes microglial activation, leading to a persistent inflammatory response (**Figure 2**).

#### 3.1.4 MiR-124

miR-124 regulates myeloid cell activity and hematopoietic function and has been found to be involved in cardiovascular diseases. In addition, miR-124 is downregulated in a wide range of human cancers. As an abundantly expressed miRNA in the brain, miR-124 promotes formation of synapses and plays an important regulatory role in many neurodegenerative diseases (62). miR-124 is specifically expressed in microglia, and miR-124 upregulation leads to microglial and CNS macrophage phenotypic switching to an anti-inflammatory state. miR-124 is an important target of many lncRNAs (63–65). Yang et al. (66) found that exogenous miR-124 promotes the transformation of microglia into an anti-inflammatory phenotype and promotes synaptic connection in the hippocampal region, as well as the recovery of neurological function after brain injury (**Table 1**, **Figure 3**). miR-124 promotes microglia transformation by inhibiting TLR4, as validated in microglia models (66). miR-124 is significantly elevated in mouse hippocampal neurons, and similar changes have been observed in the nerve tissues of AD patients, suggesting that miR-124 plays a significant role in AD nerve injury. Moreover, Wang et al. found that miR-124 affects synaptic function and consciousness disorders in AD patients by regulating protein tyrosine phosphatase non-receptor type 1 (*PTPN1)* (67). Other researchers have also found that miR-124 upregulation decreases coordination complement component 1q-like 3 (C1ql3) expression in the brains of APP/PS1 transgenic mice. Conversely, downregulation of miR-124 expression is also accompanied by changes in C1ql3, leading to Aβ deposition in the brain and cerebrovascular damage, evidenced by reductions in microvessel density and angiogenesis. Treatment of BBB damage in APP/PS1 mice with lentivirus-mediated miR-124 overexpression or C1q inhibitor (C1INH) promotes angiogenesis, reduces Aβ deposition, and, ultimately, alleviates impairments in consciousness and memory (68). Collectively, these data suggest that miR-124 regulates the neuroinflammatory response by regulating expression of complement component C1ql3 (45).

#### 3.1. 5 MiR-1199–5p

miR-1199-5p is a tumor metastasis suppressor that may be used in tumor therapy and research (69, 70). miR199 targets the RHO-associated protein kinase 1 (ROCK1) gene (**Table 1**), and ROCKs are related to cell motility and apoptosis. Alterations in miR-1199-5p levels have not been found in AD models, although research has shown that miR-1199-5p participates in regulation of the neuroinflammatory response in AD. LPS inhibits AK148321 expression in BV2 cells (71). miR-1199-5p acts as a sponge to reduce AK148321 function, and AK148321 inhibits microglia activation and reduces inflammatory cytokine release (**Figure 3**). In addition, miR-1199-5p may directly target heat-shock protein family A (HSPA5) in LPS-stimulated BV2 cells. AK148321 overexpression in BV2 cells inhibits apoptosis of hippocampal neurons, and HSPA5 downregulation prevents this inhibition (72).

#### 3.1.6 MiR-15a

miR-15a is a miRNA that is downregulated in many cancers. As a tumor suppressor gene, miR-15a loss leads to an imbalance in the expression of oncogenic bcl-2 and onco-suppressor p53 proteins (73). miR-15a is involved in regulating the inflammatory response and apoptosis and is increasingly important in neurological diseases (74). miR-15a is downregulated in the brain of AD (**Table 1**). Extracellular signal regulated kinase 1 (ERK1) is an enzyme that participates in tau phosphorylation. ERK1 expression is regulated by miR-15 in neural tissues (75). Studies on other neurological diseases have also found that miR-15 may participate in regulating neuroinflammatory responses. For example, Cai et al. (76) found that, compared with the control group, rats with chronic constriction injury (CCI) showed significantly decreased expression of miR-15a and this decrease further promoted neuroinflammation in the spinal cord tissue. Conversely, exogenous miR-15a significantly reduced the levels of CCI-induced pro-inflammatory cytokines (**Figure 3**). miR-15a also regulates protein kinase B 3 (AKT3) in a microglia model. In addition, miR-15a upregulation induces the expression of autophagy-related proteins, indicating that miR-15a is involved in AKT3-mediated autophagy *via* AKT3 inhibition. Moreover, miR-15a-5P targets NR2B in epileptic patients, negatively regulating its expression and inhibiting neuronal apoptosis in the hippocampus (77). The specific mechanism of miR-15a’s action in AD is unclear and requires further study. miR-15a may become an important target for AD.

#### 3.1.7 MiR-132

miR-132, a miRNA enriched in various tissues, is a regulatory RNA universally expressed in the cardiovascular system and specifically expressed in various cardiac diseases, for which it may serve as a potential diagnostic biomarker (78). miR-132 is involved in neuronal genesis and synaptic plasticity (79). miR-132 was found to be significantly lower in the brains of patients with AD than in normal controls (**Table 1**). Additionally, miR-132 deletion in a mouse model of AD leads to more severe Aβ deposition and more severe memory impairment when compared to AD mice with normal miR-132. Xu et al. (80) found a possible downstream target of miR-132, namely C1q, through silicon analysis. C1q, a major protein of the typical complement cascade, is highly expressed in the nerve tissue of AD patients. Further studies showed that miR-132 or C1INH treatment increases levels of synaptic protein expression when compared to controls. Moreover, subsequent studies suggested that miR-132 may regulate C1q function. Another study (81) found that miR-132 inhibits the expression of inducible NO synthase (iNOS) in the hippocampus and prevents oxidative stress *via* mitogen-activated protein kinase 1 (MAPK1) inhibition. These alterations improved the cognitive function of AD rats. miR-132 may participate in regulating the neuroinflammatory response (**Figure 3**).

miR-132 interacts other miRNAs in AD. Pascal et al. (82) found that insufficient miR-132/212 increased tau expression. Studies based on cellular genetics have shown that miR-132 regulates tau mRNA expression. Mouse models with miR-132/212 deletion exhibit greater tau protein aggregation compared to control mice. In contrast, treatment with exogenous miR-132 mimics improve partial memory function and enhance tau decomposition in AD mice. In addition, expression of miR-132 and miR-212 in the hippocampus are positively correlated with cognitive score in AD patients. These results suggest that highly expressed miR-132 and miR-212 in the brain may protect the elderly from neurological disorders (79).

#### 3.1 8 MiR-146a

miR-146a, an miRNA with abundant expression in different tissues (83), inhibits doxorubicin-induced cardiotoxicity in the myocardium (84), and its downregulation inhibits the effects of LPS on angiogenesis (85). In neural tissue studies, co-culture of miR-146a with mesenchymal stem cells has been found to reduce the astrocyte-associated inflammatory response and synaptic formation. miR-146a also improved cognitive dysfunction and memory loss in an AD mouse model (86). Further analysis showed that the levels of both miR-146a and NF-κB were decreased in the brain of AD mice treated with stem cells compared with the control mice (**Table 1**; **Figure 3**). NF-κB is an important regulator of inflammation and cancer progression (87). miR-146a has been used as a myeloid-selective NF-κB inhibitor in other diseases, such as leukemia, and has been demonstrated to inhibit NF-κB expression in AD models (30). miR-146a may participate in AD by inhibiting NF-κB and regulating the neuroinflammatory response and synaptic formation.

#### 3.1.9 MiR-223

miR-223 is expressed in a variety of tissues where it plays different roles and participates in several pathological states, such as autoimmune and inflammatory diseases (88). miR-223-3p is thought to antagonize the function of NOD-like receptor thermal protein domain associated protein 3 (NLRP3), an inflammatory microprotein (89). In myeloid bone marrow cells, downregulation of miR-223 induced NF-κB and MAPK activity, which subsequently promoted the production of inflammatory factors (90) (**Table 1**; **Figure 3**). Chronic inflammasome activation is a potential feature of neurodegenerative diseases. Studies have evaluated the serum concentrations of miR-223-3p in AD patients and healthy controls (HC) and found that miR-223-3p plasma concentrations in AD groups were reduced compared with HC (91). miR-223 may participate in AD by affecting the neuroinflammatory response, making it a potential therapeutic target.

#### 3.1.10 MiR-125b

mir-125b is an important cancer regulator that acts through multiple signaling pathways, such as the Wnt, PI3K/Akt, STAT-3, MAPK, NF-κB and p53 pathways (92). Moreover, miR-125b is an important inflammation-related miRNA that is richly expressed in neural tissues (93). miR-125b upregulation induces tau deposition in rat brain, whereas inhibition of miR-125b expression targets beta amyloid cleaving enzyme 1 (BACE1) to reduce apoptosis and oxidative stress, thereby inhibiting Aβ-induced neurotoxicity (94) (**Table 1**). miR-125b is increased in AD patients (95). Moreover, miR-125b overexpression inhibits the proliferation of AD model cells *in vitro*, induces apoptosis, and enhances inflammation and oxidative stress (96) (**Figure 3**). On the contrary, IL-10 activity is significantly reduced in *in vitro* models of AD (97). Therefore, the proinflammatory microRNA, miR-125b, may promote development of AD.

#### 3.1.11 MiR-181a

The target genes of miR-181a contain multiple apoptosis-related genes, and dysregulation of miR-181a expression is closely related to the occurrence and development of a variety of cancers. miR-181a is abundant in neural tissues and affects synaptic regeneration and functional repair (83, 98). It has previously been reported that miR-181a is upregulated in the hippocampus of an AD mouse model (**Table 1**; **Figure 3**). Inhibition of miR-181a in this AD mouse model reduced memory deficits and increased glutamic acid (GluA) 2 and GluA1 levels, which regulate cognitive impairment. miR-181a is expressed in T cells, where it regulates their activation threshold (99). Interestingly, miR-181a has been verified as being involved in age-related diseases, as the expression of miR-181a in naive T cells decreases with age (100). Although the exact mechanism is unclear, miR-181a likely participates in regulating the AD neuroinflammatory response.

There are many other miRNAs that have been implicated in regulating inflammatory responses, including miR-150, miR-21, and Let-7 (**Figure 3**). These same miRNAs may be involved in AD-related neuroinflammation. For example, miR-150 is downregulated in AD patients (**Table 1**), and miR-150 knockdown enhances the activity of hippocampal neurons in a model of consciousness disorder. Conversely, miR-150 overexpression significantly increases apoptosis in the PC12 cell line (101). In addition, miR-150 was found to influence the formation of mature B cells. miR-150 is abundant in lymphocytes, and its expression changes with differentiation and maturation of B cells (102). miR-150 may be involved in regulating the inflammatory response *via* B cell regulation (**Figure 3**). miR-21 is an anti-inflammatory regulatory factor that is abundant in microglia (103, 104) regulatory factor that is abundant in microglia (105). miR-21 expression is induced by MyD88 and NF-κB, resulting in NF-κB downregulation (106). In addition, miR-21 reduces TNF-α secretion and, thus, promotes macrophage switching to an anti-inflammatory phenotype and inhibits microglial activation (107). Let-7, an important regulator of neuroinflammation, is an evolutionarily conserved family of miRNAs (108, 109). Let-7 affects TLR-7 function to regulate the neuroinflammatory response, making it a potential regulator of AD. Although an increasing number of miRNAs are being discovered and studied, the role of many miRNAs remains unknown.

### 3.2 LncRNAs in AD

lncRNAs are larger than 200 nt, ranging up to 100 kb in size. lncRNAs participate in many important physiological processes (110) and regulate protein formation at the transcriptional and post-transcriptional levels. Further, lncRNAs interact with other RNAs as part of the RNA network. For example, lncRNAs act as miRNA sponges to regulate miRNA functions, and, in turn, miRNAs regulate lncRNA function, activation, and stability. lncRNA is highly expressed in mammalian neural tissue, enabling it to respond quickly to environmental and molecular changes. In addition, lncRNAs participate in the physiological processes of a variety of neurodegenerative diseases such as AD, Parkinson’s disease, and Huntington’s disease (111–113). Neuroinflammation is an important factor driving deterioration in various neurodegenerative diseases. With disease progression, inflammatory cells are constantly stimulated, and the formation of persistent neuroinflammation can cause damage to the nerve tissue. Many studies have shown that lncRNAs participate in various neuroinflammatory processes.

#### 3.2.1 LncRNA MALAT1

**Figure 4:**
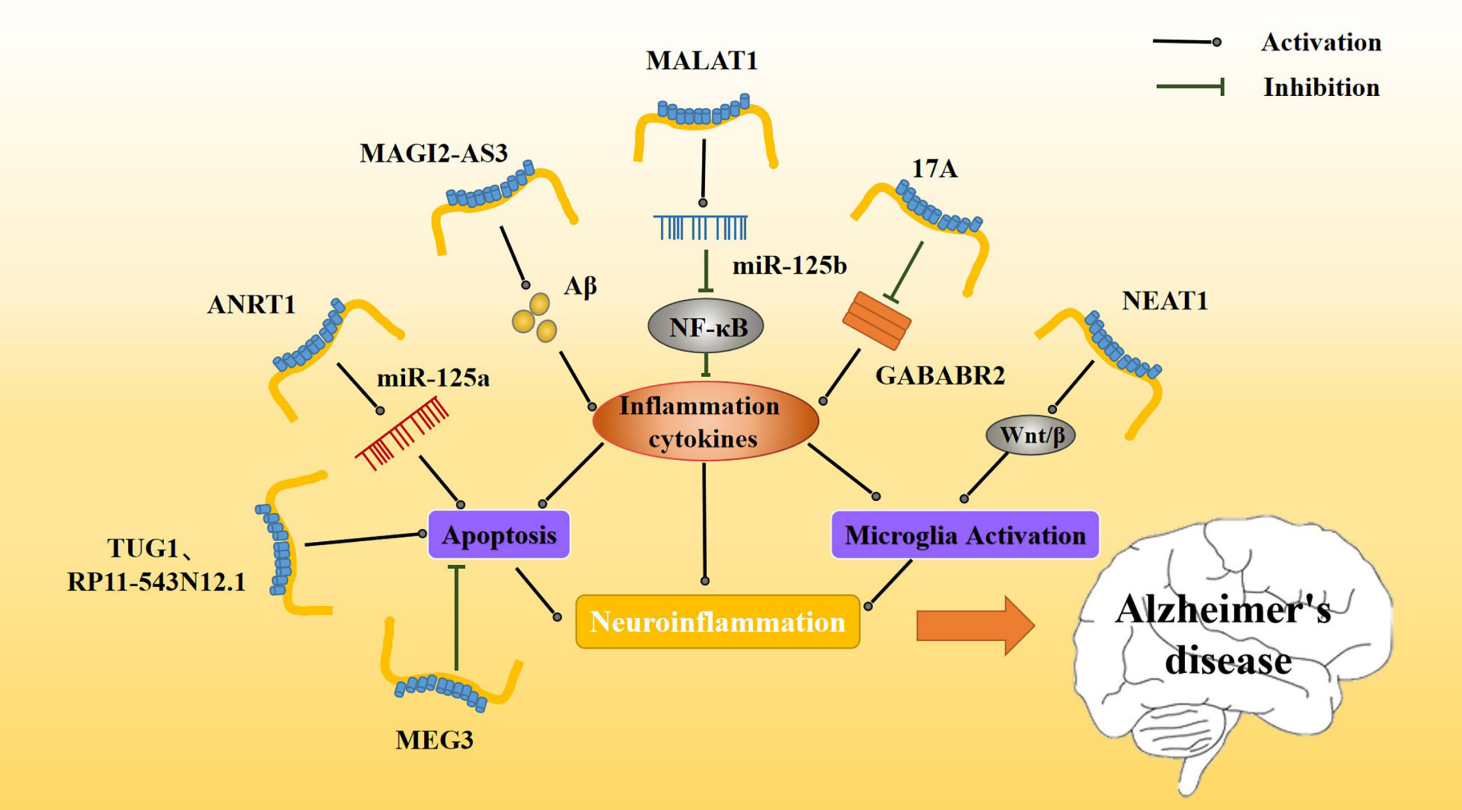
Effect of inflammatory lncRNAs on AD inflammation. Multiple inflammatory lncRNAs may be involved in different inflammatory pathways. MAGI2-AS3 promotes the Aβ-induced inflammatory response, whereas MALAT1 regulates release of inflammatory cytokines *via* NF-κB. The release of inflammatory mediators further affects the activation and apoptosis of microglia. Together, these constitute a persistent neuroinflammatory response that accelerates the progression of AD.

Metastasis-associated transcript 1 (MALAT1) was first identified in human lung adenocarcinoma and then found to be expressed in a variety of tissues. MALAT1 plays an important role in regulating cellular proliferation, apoptosis, autophagy, and other physiological processes (114). Upregulation of MALAT1 inhibits neuronal apoptosis and neuroinflammation in patients with AD. Some scholars believe that the mechanism of MALATI-induced inhibition may be related to microglia activation *via* NF-κB inhibition. Peizhi et al. (97) found that in an AD mouse model, MALAT1 upregulation reduced neuronal apoptosis, promoted neuronal functional repair and regeneration, and downregulated IL-6 and TNF-α levels (**Figure 4**) while upregulating IL-10 levels compared to control animals. Downregulation of MALAT1 produced the opposite effects. Additionally, MALAT1 was found to inhibit miR-125b expression. In an AD model overexpressing MALAT1, miR-125b was downregulated and this downregulation was associated with reduced inflammatory cytokine release.

#### 3.2.2 LncRNA NEAT1

Abnormal overexpression of long non-coding RNA nuclear paraspeckle assembly transcript 1 (NEAT1) has been found in several types of solid tumors, such as lung cancer, colorectal cancer, and hepatocellular carcinoma. As an important nuclear gene, NEAT1 is closely related to physiological processes, such as cell replication, growth, and apoptosis (115). NEAT1 is upregulated in the nerve tissue of AD patients (**Table 1**), and it has been found that amyloid deposition in AD patients causes NEAT1 upregulation. Upregulation of NEAT1 also affects AD progression by increasing the apoptosis rate and decreasing the activity of nerve cells. Moreover, NEAT1 upregulation increases expression of amyloid, BACE1, APP, and tau and increases phosphorylation levels of these proteins. It also increases caspase 3. Increases in these proteins or in their phosphorylation levels may be related to NEAT1 triggering neuroinflammation (116). A number of experiments have shown that NEAT1 activates microglia, promotes neuroinflammation, upregulates inflammatory mediators such as IL-2 and IL-6, and promotes the progression of various neurological diseases (**Figure 4**). NEAT1 may contribute to neuroinflammatory injury through the Wnt/β signaling pathway during brain hypoxia injury (117).

#### 3.2.3 LncRNA RP11-543N12.1

lncRNA RP11-543N12.1 is highly expressed in AD patients (**Table 1**). RP11-543N12.1 expression is higher in the nucleus than in the cytoplasm, suggesting that RP11-543N12.1 may regulate transcription in the nucleus. In addition, RP11-543N12.1 and Aβ25-35 synergistically promote miR-324-3p expression and promote cell apoptosis during AD progression (**Figure 4**). Upregulation of RP11-543N12.1 and miR-324-3p may cause cellular apoptosis by promoting continuous release of inflammation-related factors. lncRNA RP11-543N12.1 acts as a miR-324–3p sponge in microglia and neuron models, promoting neuroinflammation and tau deposition and phosphorylated (118).

#### 3.2.4 LncRNA MEG3

Maternally expressed gene 3 (MEG3) was first found to be an important tumor suppressor, specifically expressed in lung cancer and hepatocellular carcinoma. MEG3 inhibits the biological functions of tumor cells by regulating major tumor suppressor genes p53 and Rb (119). lncRNA MEG3 expression is decreased in AD patients (120) (**Table 1**). In addition, upregulation of MEG3 in AD rat models improves AD prognosis, reduces memory loss, inhibits neuronal injury and apoptosis, and reduces Aβ deposition. The mechanism of lncRNA MEG3 action is believed to be related to a neuroinflammatory response to MEG3. Jiping et al. (121) reported that MEG3 upregulation is associated with decreased levels of apoptosis (**Figure 4**), PI3/Akt signaling pathway-related proteins, and inflammation-related proteins. MEG3 is likely to play a neuroprotective role by inactivating the PI3/Akt pathway to decrease oxidative damage and inflammation.

#### 3.2.5 LncRNA ANRIL

lncRNA at the INK4 locus (ANRIL), a newly discovered lncRNA, is closely associated with regulation of cell growth and apoptosis in various cancers, and has also been associated with vascular endothelial injury in cardiovascular diseases (122). ANRIL is associated with many inflammatory and neurological disorders, including various ischemic injury diseases, and may participate in regulating the inflammatory response *in vivo* (123, 124). Recently, lncANRIL was found to be upregulated in the brain of AD patients (**Table 1**), and this upregulation may also be related to the progression of the AD neuroinflammatory response. In an AD model, lncANRIL binds mir-125a. In terms of inflammatory response, lncANRIL downregulation reduces the production of many inflammatory mediators, such as TNF-α and IL-1β, and inhibits apoptosis. In addition, upregulation of lncANRIL increases neuronal growth and miR-125a expression, whereas miR-125a inhibition weakens the neuroprotective effect of lncANRIL downregulation in cellular AD models (**Figure 4**). Luciferase reporter gene detection has shown that lncANRIL directly binds to miR-125a in an AD mouse model (125). lncANRIL may interfere with neuroinflammatory responses by acting on miR-125a. This specific mechanism requires further study and may represent a possible new breakthrough for AD and other inflammatory diseases in the future.

#### 3.2.6 LncRNA 17A

lncRNA 17A is upregulated in the brain of AD patients (126) (**Table 1**), and it may affect the progression and prognosis of AD by inducing formation of Aβ. The proportion of apoptotic cells decreases when 17A is inhibited and increases when 17A is overexpressed. Studies have found that 17A blocks GABAB2, affecting cAMP production and K(+) channel activation to inhibit its signal transduction (127). The expression of 17A in different cells is associated with GABAB receptor (GABABR) 2 expression. Specifically, when 17A is overexpressed, GABABR2 expression is downregulated (**Figure 4**). The GABABR is involved in regulating microglia function and neuroinflammation, and GABABR2 is involved in the inhibition of synaptic transmission. It has been reported that upregulation of GABABR2 inhibits the release of pro-inflammatory cytokines. 17A may participate in AD by regulating neuroinflammation *via* GABABR (128). Recently, it was found that the role of 17A in determining AD progression may also be regulated by Wnt/β-catenin (126). The mechanism whereby 17A regulates neuroinflammation requires additional study, as it has the potential to become a novel target for AD.

#### 3.2.7 LncRNA MAGI2-AS3

MAGI2-AS3 is a lncRNA transcribed from the antisense strand, near the MAGI2 gene. MAGI2-AS3 plays an important regulatory role in many cancers, including lung cancer (129–132). MAGI2-AS3 also acts as a miR-374b-5p sponge in many cancers (133, 134). MAGI2-AS3 is upregulated in patients with AD. MAGI2-AS3 acts as a sponge affecting the function of miR-374b-5p and BACE1, an enzyme important for degrading Aβ protein. In addition, some scholars have found that MAGI2-AS3 may play a regulatory role in chronic inflammation (**Table 1**, **Figure 4**). Studies have shown that upregulation of MAGI2-AS3 promotes Aβ deposition and inflammation, leading to speculation that MAGI2-AS3 may be related to the neuroinflammatory response in AD. Moreover, it downregulation of MAGI2-AS3 enhances neuronal activity and reduces neuroinflammation, and miR-374b-5P overexpression causes similar results (135). The MAGI2-AS3/miR-374b-5p axis may be a novel target for the treatment for AD.

#### 3.2.8 LncRNA TUG1

lncRNA taurine upregulated gene 1 (*TUG1*) (136), a novel lncRNA, participates in ischemic disorders, including ischemic spinal cord injury and ischemic myocardial injury (137, 138). It also promotes neuronal apoptosis through the absorption of miR-9 by sponges. It was found that small interference TUG1 (Si-TUG1) mitigates neuroinflammation in ketamine-induced hippocampal neurons in rats. Hence, some scholars believe that TUG1 may also regulate neuroinflammation in AD (**Table 1**). Li et al. found that TUG1 silencing and miR-15a up-regulation alleviated memory loss, improved pathological damage, inhibited apoptosis, and enhanced antioxidant capacity of the nerve tissue in an AD rat model (139). In an *in vitro* model, hippocampal neurons treated with Aβ_25-35_ exhibited inhibition of neuronal activity and enhanced apoptosis (**Figure 4**). TUG1 silencing and miR-15a upregulation inhibit the activity of Aβ, but the underlying mechanism for these results needs further study.

### 3.3 CircRNAs in AD

circRNA is a type of RNA molecule characterized by a single stranded ring, found in a variety of species from lower to higher organisms (140). circRNA effectively prevents transcription of microRNAs, and through this microRNA transcription regulation, it affects downstream mRNA expression (141, 142). In addition, emerging evidence suggests that circRNA participates in various protein modifications. circRNAs are formed in higher eukaryotes by reverse splicing loops of pre-mRNA, creating a covalent linkage between the downstream 5’ end of the exon and the upstream 3’ end. circRNAs are resistant to exonuclease degradation due to the 5’ and 3’ linkages and are, therefore, more stable than linear mRNAs (with a half-life of greater than 48 h). circRNAs exhibit a variety of physiological functions, including acting as miRNAs and protein sponges, regulating parental gene transcription, and participating in mRNA splicing. circRNAs play a role in AD, heart failure, and hypertrophy. They are also abundant in neural tissues and are involved in the production of inflammatory factors in physiological and pathophysiological processes. For example, circ_0000950 acts as an miR-103 sponge to stabilize mir-103 function and increase the inflammatory response. CIRS-7, circ NF1-419, circHDAC9, and circAβ-a have also been confirmed to be involved in the pathogenesis of AD, with specific changes observed in AD patients. However, the potential role of circRNAs in AD is still largely unknown.

#### 3.3.1 Circ_0000950

**Figure 5:**
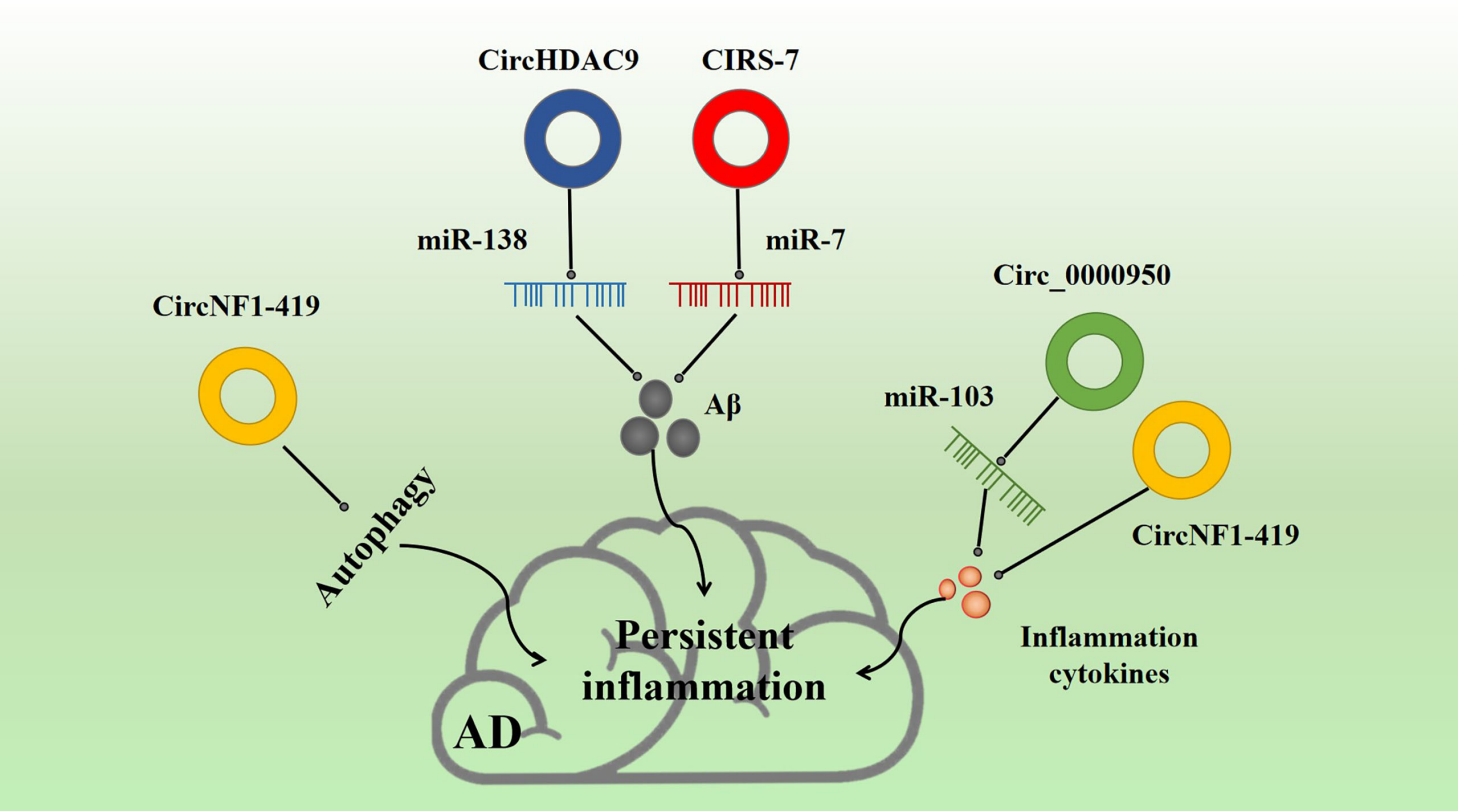
Effect of inflammatory circRNA on AD inflammation. Multiple inflammatory circRNAs participate in various inflammation-related pathways. For example, CIRS-7 and NF1-419 promote Aβ-induced inflammation. circ_0000950 and circNF1-419 are associated with the release of inflammation cytokines, and circNF1-419 is associated with autophagy.

circ_0000950 is overexpressed in AD patients (**Table 1**). The circRNAs act as a sponge for miR-103 and may regulate lipid metabolism through miR-103. Yang et al. found that circ_0000950 overexpression promoted neuronal apoptosis, inhibited neuronal growth, and increased the levels of many inflammatory factors, such as Il-1β and TNF-α in an AD model (**Figure 5**). Luciferase assay confirmed that circ_0000950 achieves its effects by direct sponge absorption of miR-103 (143).

#### 3.3.2 CIRS-7

CIRS-7 is a common endogenous circRNA found in many tissues. Hansen et al. (144) proved for the first time in 2013 that CIRS-7 acts as a miR-7 sponge, regulating gene translation and protein modification. In particular, CIRS-7 can regulate cancer progression through competitive inhibition of miR-7 (145), which has been proven to be related to the occurrence and development of renal cell cancer (146), cervical cancer (147), oral cell cancer (148), and thyroid cancer (149). Moreover, CIRS-7, as a miR-7 sponge, is involved in determining BACE1 and APP concentrations by promoting proteasome and lysosomal degradation of BACE1 and APP. Thus, CIRS-7 is a major regulatory factor in the progression of AD (150) (**Table 1**, **Figure 5**). CIRS-7 overexpression reduces Aβ deposition, suggesting that circRNAs may possess a neuroprotective function. CIRS-7 inhibits NF-κB, a key regulatory target of inflammation. CIRS-7 has also been implicated in regulating inflammation, and the CIRS-7/miR-7 axis may play a role in inducing apoptosis and inflammation *via* IL-1β (151). CIRS-7 has also been found to promote autophagy and inflammation in various neurological diseases (152).

#### 3.3.3 CircNF1-419

circNF1-419 is involved in tumor development and downregulated in AD (**Table 1**) (153). In mouse astrocytes, it has been shown that NF1-419 regulates astrocyte autophagy through the PI3K-Akt-MTOR signaling pathway. circNF1-419 overexpression in the mouse brain promotes autophagy by binding AP2B1. This interaction regulates the aging biomarkers P21, P35/25, and P16 and the pro-inflammatory factors TNF-α and NF-κB, and leads to downregulation of tau, P-tau, Aβ-1, and APOE, thereby delaying the onset of AD (154, 155) (**Figure 5**).

#### 3.3.4 CircHDAC9

circHDAC9 is downregulated in parallel to miR-138 upregulation in AD (**Table 1**) (156). miR-138 plays a tumor suppressive role by targeting genes that regulate the proliferation and apoptosis of tumor cells, thus affecting the development and prognosis of cancer. circHDAC9 binds mir-138 to produce similar effects. Moreover, circHDAC9 enhances Sirt1 inhibition and reduces miR-138-related Aβ deposition. More importantly, circHDAC9 expression is downregulated in both AD and MCI patients. Moreover, Aβ_42_ has been found to induce significant downregulation of circHDAC9 and overexpression of miR-142-5p in neurons (**Figure 5**). In addition, circHDAC9 acts as a sponge for miR-142-5p. Upregulation of circHDAC9 mitigates Aβ_42_-induced neuroinflammation and neurotoxicity in HN cells *via* miR-142-5p (157).

Other circRNAs such as circRNA-017963 also participate in the pathological development of AD. Huang et al. (158) found that the expression of circRNA-017963 was greatly decreased in 10-month-old SAMP8 mice, suggesting that circRNA-017963 may have a significant role in AD. Furthermore, miR-7033-3p may show the greatest relationship with circRNA-017963 and is likely to be involved in autophagosome formation and synaptic transmission, which have been proven to be related to AD pathogenesis.

### 3.4 PiRNAs in AD

Piwi-interacting RNAs (piRNAs) have a length of 24–30 nt and represent a unique class of RNA that are important in governing stem cell (SC) function (159, 160). piRNA is not conserved among species, and there is a considerable difference in its biological origin when compared to miRNAs and siRNAs (161). The piRNA source is not dsRNA, so Dicer shearing is not involved. However, mature piRNAs can target transposers in the genome and silence them (162). piRNAs are also involved in regulating epigenetic function and stabilizing mRNA. In general, piRNA is highly expressed in reproductive tissues and acts as a guardian of the genome through stable transposons.

More recently, however, piRNA has been found in other tissues, including nerve tissue (163), suggesting that it may be involved in neuro-related diseases. Kim et al. have reported that the piwi-piRNA pathway may govern neuronal function in many animals, affecting axonal regeneration and memory loss (164). In addition, there is evidence that the piwi/piRNA complex promotes synaptic repair and reduces memory loss *via* cAMP response element-binding protein (CREB) (165). Analysis of piRNAs in cerebrospinal fluid exosomes may improve the antecedent diagnosis of AD and may also help with patient stratification in clinical trials. From a therapeutic perspective, it is of importance to find a biomarker that helps predict the transition to AD dementia in patients with MCI (166). In addition, piRNAs have been shown to be important for maintaining genomic integrity and inhibiting transposer activity, and piRNAs may be involved in the origination of single nucleotide polymorphisms (SNPs) associated with AD (167). With further research on piRNA still ongoing, the mechanism of its involvement in AD will become clearer; although extensive research is still needed.

### 3.5 TsRNAs in AD

Transfer RNA-derived small RNAs (tsRNAs) can be divided into two types based on biological origin: tRNA-derived fragments (tRFs) and half of tRNA (tiRNAs) (168, 169). The length of tRF ranges from 14 to 36 nt. tRFs can be divided into tRF-1, tRF-2, tRF-3, tRF-5, and i-tRF, according to their cleavage site and origin. The formation of tiRNAs involves the specific cleavage of angiopoietin on the anticodon ring of mature tRNA. These tsRNAs regulate a range of molecular processes, from gene translation and RNA stability to cellular proliferation and differentiation (170, 171).tsRNA is a new RNA species found abundantly in many tissues including neural tissue (172). Although it is largely unstudied in the context of neurological diseases, Magistri et al. (173) demonstrated that the upregulation of try-tRNA and arg-tRNA is involved in altering RNA kinase in CLP1 knockout mice, leading to the gradual loss of motor nerve function in affected mice. Shortly thereafter, another study found that accumulation of tRNA fragments caused by tRNA processing defects may lead to the development of neurodegenerative diseases (174). These tsRNAs make motor neurons more susceptible to oxidative stress, indicating that tsRNAs may be involved in neurodegenerative diseases *via* neuronal oxidative stress response regulation. The TRF expression from tRNA subsets is altered in the hippocampus of AD patients. Changes in TRF expression may be related to age and disease stage. Decreased NOP2/Sun RNA methyltransferase 2 (NSun2) expression has been observed in AD patients aged <65 years, suggesting that decreases in NSun2 may reduce the degree of tRNA methylation to enhance tRF production (175). As a newly discovered sncRNA, many mechanisms of tsRNA action in the human body are still unclear. In the future, they may become important targets for AD.

In conclusion, comprehensive investigations of non-coding RNAs, such as miRNAs, lncRNAs, circRNAs, piRNA, and tsRNA, that includes interactions with other RNAs, DNAs, and proteins, will be helpful in fully elucidating the cause/consequence relationships of AD dysfunction. It is of significant importance to develop a valid therapy to treat this devastating disease.

## 4 Potential Therapeutic Targets

There is no doubt that ncRNAs play a significant role in several neuroinflammatory diseases (37). For many diseases with unclear molecular mechanisms of pathogenesis and progression, ncRNAs may serve as therapeutic targets in the future (120, 176). In fact, ncRNA-based therapies have been developed for many diseases with complex mechanisms that are difficult to cure, such as various cardiomyopathies, diabetes, and neurodegenerative diseases (177, 178). Thus, many novel strategies have been invented to control the function of ncRNAs. Oligonucleotides have several advantages in targeting ncRNAs in contrast to traditional therapies (e.g., small molecules and antibodies) (179). Oligonucleotides complement their target RNA precisely, making screening and detection of targeting tools relatively simple. In addition, oligonucleotides can enter cells and specifically target RNAs that are inaccessible to many factors acting on cell membranes (180, 181). Because oligonucleotides are more targeted than other subclasses of drugs, potential side effects are reduced (182).

Abnormalities in the modulation of miRNA expression have been observed in a large number of diseases and are regarded as feasible therapeutic targets and regulators with a wide variety of applications for preclinical and clinical trials (183–185). miRNA mimics represent a novel approach to miRNA therapy. miRNA mimics are small double-stranded RNA molecules derived from the exogenous synthesis of relevant miRNAs that are modified into functional miRNAs (186). Other miRNA mimics may act to inhibit the function of endogenous miRNAs, and their synthesis is usually based on the complementary sequence of the relevant miRNA. These miRNA mimics block the binding of endogenous miRNAs to their target and include anti-antibodies, miRNA sponges, and small molecule antisense oligonucleotides (187).

Current therapies targeting lncRNAs include oligonucleotide compounds (e.g., thiocarcinogenic main chains and various sugar modifications). Targeting of lncRNAs can also be achieved by blocking or knocking out their expression *via* antisense oligonucleotides (ASOs) or small interfering RNA (siRNA) (120, 188). These methods have been tested clinically with promising results. CRISPR-Cas9 is an effective tool for gene mapping and modification (189). Cas9 endonucleases can be targeted for DNA editing or gene knockout. In a novel modification to the approach, Cas9 binds to transcriptional activators such as NF-κB to upregulate genes. Crispr-cas9-based therapies are already used to treat some autoimmune diseases such as cystic fibrosis (190).

The major obstacle in AD treatment is providing ncRNA-based therapies that can cross the BBB (191). Promising strategies currently under investigation include using non-viral pathways and lipid or polymer nanoparticle delivery systems (192, 193). Focused ultrasound is another technique that has been found to temporarily block the BBB, promising the application of targeted therapy in brain tissue (25, 194). Adeno-associated virus (AAV) vectors have been shown to target ncRNAs in the CNS (195, 196). Therefore, identifying ideal ncRNA-targeted drugs may help improve the management and treatment of pathologic neuroinflammation. The discovery of ncRNAs provides new perspectives for understanding genetic treatments (176). The promise of ncRNAs is that they affect multiple genes or biological processes, although attempting to understand their full potential is not without challenges.

## 5 Conclusions

Dysregulation of neuroinflammation-related ncRNAs leads to dysfunction of certain signaling pathways *via* regulation of target genes that act as inflammatory pathologic factors in the development and progression of AD. As described above, these ncRNAs regulate key genes that may play a significant role in the development of AD. Existing studies have shown that these ncRNAs influence the progression of AD in cell and animal models and in human patients. Thus, by studying the physicochemical properties of these ncRNAs and their specific mechanisms related to disease, effective treatments for AD may be discovered. For example, induction of anti-inflammatory ncRNAs or inhibition of pro-inflammatory ncRNAs may represent a therapeutic strategy to improve central nervous system tissue damage following neuroinflammation (197–199). Oligonucleotide therapy is an emerging tool for ncRNA targeting and has the benefits of specific targeting and low toxicity. Thus, oligonucleotide therapy is currently the most widely researched method in ncRNA-targeting drug discovery and application (182). Despite advances in determining how ncRNAs act in neuroinflammation, the characteristics and role of many ncRNAs remain unknown.

There are limitations that need to be addressed. First, most ncRNAs are found in low concentrations in bodily fluids, and the ncRNA extraction and separation methods are complicated. Moreover, owing to the easily degradable nature of ncRNAs, it is difficult to ensure the quality of preservation. Second, the four existing ncRNA detection methods all have inherent defects. For example, it is difficult to construct cDNA libraries for ncRNA molecules with low expression levels. In addition, the results of qRT-PCR are uncertain, making primer design crucial. The reproducibility of gene chip technology is poor, and the results are closely related to the sample size. Finally, high-throughput sequencing is costly and wasteful. Further research is needed to develop accurate diagnostic criteria for clinical use. With more extensive research, the understanding of the mechanisms of ncRNA will become more substantial, and it will be an important means for the research and treatment of AD.

Although significant advances have been made in the physiological characteristics of ncRNA and its role in neuroinflammation associated with AD, this field remains largely unknown. Future investigation should give top priority to targets and biological processes that are involved in the key processes of AD pathology, validate them in model organisms that are more closely related to humans, and develop new experimental paradigms to improve research efficiency. Because the ultimate goal of these studies is to prevent the occurrence of AD or improve the prognosis of AD patients, differences in clinical responses based on patient age and gender are also crucial for the study of AD. It is important that this effort be combined with more attention to clinical application in the clinical transformation of AD.

## Author Contributions

Conceiving, YL, ZZ, YX, and WP. Searching literature, YL, XC, SH, and ZZ. Manuscript drafting, YL, XC, HL, and SH. Manuscript revising, YL, HL, ZZ, YX, and WP. All authors contributed to the article and approved the submitted version.

## Funding

This work was financially supported by the National Natural Science Foundation of China (No. 81873169, 82104967), Hunan Provincial Natural Science Foundation of China (No. 2020JJ4803), Hunan Scientific Research Program of Traditional Chinese Medicine (No.2021192).

## Conflict of Interest

The authors declare that the research was conducted in the absence of any commercial or financial relationships that could be construed as a potential conflict of interest.

## Publisher’s Note

All claims expressed in this article are solely those of the authors and do not necessarily represent those of their affiliated organizations, or those of the publisher, the editors and the reviewers. Any product that may be evaluated in this article, or claim that may be made by its manufacturer, is not guaranteed or endorsed by the publisher.

## Glossary

**Table:**

Aβ	beta-amyloid
AD	Alzheimer’s disease
AICD	intracellular binding domain of amyloid precursor protein
*AnkG*	ankyrin-G
ANRIL	LncRNA at the INK4 locus
*AP2β1*	activating enhancer binding protein 2 beta 1
APP	amyloid precursor protein
BACE 1	beta amyloid cleaving enzyme 1
BBB	blood-brain barrier
ncRNA	non-coding RNA
C1ql3	complement component 1q-like 3
CCI	chronic constriction injury
circRNA	circular RNA
*CREB*	cAMP response element-binding protein
*ERK1*	extracellular signal regulated kinase 1
GABABR	gamma aminobutyric acid B receptor
GluA	glutamic acid
HSPA5	heat-shock protein family A
IFN-β	interferion-β
iNOS	inducible NO synthase
LncRNA	long non-coding RNA
LPS	lipopolysaccharides
miRNA	microRNA
*MAGI*	Membrane-associated guanylate kinase with an inverted arrangement of protein-protein interaction domains
MAGI2-AS3	MAGI2 antisense RNA 3
MAPK1	mitogen-activated protein kinase 1
*MALAT1*	metastasis-associated transcript 1
*MEG3*	maternally expressed gene 3
MCI	mild cognitive impairment
mTOR	mammalian target of rapamycin
MyD88	myeloid differentiation factor 88
NEAT1	nuclear paraspeckle assembly transcript 1
NET	neutrophil extracellular trap
NF-кB	nuclear factor-kappa B
NLRP3	NOD-like receptor thermal protein domain associated protein 3
NO	nitric oxide
PAD4	peptidylarginine deiminase type4
PI3K	phosphatidylinositol-3-kinase
Akt	protein kinase B
piRNA	PIWI-interacting RNA
PTGS2	prostaglandin Endoperoxide Synthase 2
*PTPN1*	protein tyrosine phosphatase non-receptor type 1
*ROCK1*	Rho-associated protein kinase 1
*SIRT1*	silent mating type information regulation 2 homolog 1
*SOCS-1*	suppressor of cytokine signaling 1
*SphK1*	Sphingosine Kinase 1
*SYT1*	synaptotagmin1
TLR	toll-like receptor
TNF	tumor necrosis factor
TNFR1	tumor necrosis factor receptor 1
TREM2	triggering receptor expressed on myeloid cells 2
tsRNA	transfer RNA-derived small RNA
*TUG1*	taurine up-regulated gene 1
3’UTR	3’untranslated region
